# Immune Curbing of Cancer Stem Cells by CTLs Directed to NANOG

**DOI:** 10.3389/fimmu.2018.01412

**Published:** 2018-06-19

**Authors:** Christina Wefers, Gerty Schreibelt, Leon F. A. G. Massuger, I. Jolanda M. de Vries, Ruurd Torensma

**Affiliations:** ^1^Department of Tumor Immunology, Radboud Institute for Molecular Life Sciences, Radboudumc, Nijmegen, Netherlands; ^2^Department of Obstetrics and Gynecology, Radboudumc, Nijmegen, Netherlands

**Keywords:** dendritic cell vaccination, cancer stem cells, MHC class I, NANOG, cancer stem cell immunity

## Abstract

Cancer stem cells (CSCs) have been identified as the source of tumor growth and disease recurrence. Eradication of CSCs is thus essential to achieve durable responses, but CSCs are resistant to current anti-tumor therapies. Novel therapeutic approaches that specifically target CSCs will, therefore, be crucial to improve patient outcome. Immunotherapies, which boost the body’s own immune system to eliminate cancerous cells, could be an alternative approach to target CSCs. Vaccines of dendritic cells (DCs) loaded with tumor antigens can evoke highly specific anti-tumor T cell responses. Importantly, DC vaccination also promotes immunological memory formation, paving the way for long-term cancer control. Here, we propose a DC vaccination that specifically targets CSCs. DCs loaded with NANOG peptides, a protein required for maintaining stem cell properties, could evoke a potent anti-tumor immune response against CSCs. We hypothesize that the resulting immunological memory will also control newly formed CSCs, thereby preventing disease recurrence.

## Current Approaches to Target Cancer Stem Cells (CSCs)

CSCs form a rare population of tumor cells with a unique ability to self-renew. Although CSCs proliferate slowly, they give rise to more differentiated, fast-growing tumor cells that sustain and fuel tumor growth. Their low proliferation rate, combined with the expression of drug transporters, enables CSCs to survive classical anti-tumor treatments. Furthermore, continuous genomic and epigenomic changes allow CSC to develop new resistance mechanisms that can be passed on to their progeny (1). Current anti-tumor therapies targeting the tumor bulk thus often fail to eliminate CSCs.

The realization that eradicating CSCs is essential to prevent disease relapse has greatly stimulated CSC research. The search term “cancer stem cells” yields over 75,000 hits in PUBMED, and numerous papers describe CSC proteins and their pathways. This has lead to different treatment strategies that target CSC, either by inducing CSC killing, by forcing their differentiation, or by inhibiting key signaling pathways.

Initially, large libraries of small molecules were screened for candidates that kill CSCs [for a recent review see Ref. (2, 3)]. These molecules target different key signaling pathways of CSCs. Salinomycin was one of the first compounds discovered (3). Another example is the small molecule IGC-001 which affects the Wnt pathway by interfering with β-catenin (4). NOTCH signaling could be inhibited through γ-secretase inhibition of MK-0752 (5). The two FDA approved drugs Vismodegib and Erismodegib block the Hedgehog signaling pathway and are tested in clinical trials for the treatment of basal-cell carcinoma (6–8).

Aside from synthetic molecules, also naturally occurring small molecules and food components can interfere with CSC signaling. A vast number of papers describe the signaling targets of these natural compounds and the mechanisms of CSCs killing [for review see Ref. (9)]. Several compounds showed inhibiting functions *in vitro* (10, 11). Statistical analyses even demonstrated that eating cruciferous vegetables (12, 13) can prolong survival of cancer patients. Curcumin is effective in several tumors. However, curcumin only works at high dosage (8 g/day), probably because its low solubility limits availability. A modified, more soluble form of curcumin is, therefore, being tested in several trials (14). Although food components killed tumor cells *in vitro*, they could not prevent cancer development *in vivo* (15–17). A likely reason for this discrepancy is that food mostly contains inactive precursors of active compounds. For example, only a minority of people has an intestinal flora that promotes the conversion of the precursor glucoraphanin into the CSCs inhibitor sulforaphane (18, 19).

Just like their synthetic counterparts, natural small molecules from food components affect the Hedgehog-, the Wnt-, and the Notch-Jagged signaling pathways. However, this approach can result in severe side effects, as these signaling pathways are also essential for normal stem cells. Stem cells in the colon crypts—which are crucial for regenerating and sustaining colon tissue—depend on the Wnt pathway. Targeting Wnt signaling, therefore, comes with a risk of collateral damage (20–22).

To prevent these side effects, antibody–drug conjugates (ADCs) can be used to specifically target and kill CSCs *via* cell surface markers, such as LGR5, CD133, or DLL3 (23–25). Even though these ADCs showed promising results in murine experimental models of colon and lung cancer, their success should be interpreted with caution. CSC markers are heterogeneously expressed on the stem cell population, and to date, none of the identified surface markers is specific for CSCs (26). Aspecific ADCs may also eradicate normal stem cells that share surface markers with CSCs. Furthermore, the instability of current ADCs in the circulation may lead to premature drug release and off-target toxicity (27).

Another approach induces terminal differentiation of CSCs through epigenetic targeting. The best-known example is all-trans retinoic acid, which is used to treat acute promyelocytic leukemia. This compound induces histone modifications that force CSCs to differentiate (28). Similarly, histone deacetylases (HDAC) are promising targets in CSCs, as several clinically available HDAC inhibitors can preferentially target CSCs *in vitro* (29). However, little is known about the epigenetic regulation of CSC and treatment with HDAC inhibitors could cause toxicity by disrupting gene regulation in normal tissue stem cells.

Even though current approaches to target CSCs in solid tumors are promising, they do face major challenges. First, reliable CSC-specific markers and signaling pathways need to be identified to prevent off-target effects. Second, none of these strategies can cope with CSC plasticity, the interconversion of CSCs and more differentiated tumor cells. Eradication of CSCs can only be achieved if these problems are adequately addressed.

## Stem Cell Transcription Factors are Ideal Targets to Inhibit CSCs

**Figure 1:**
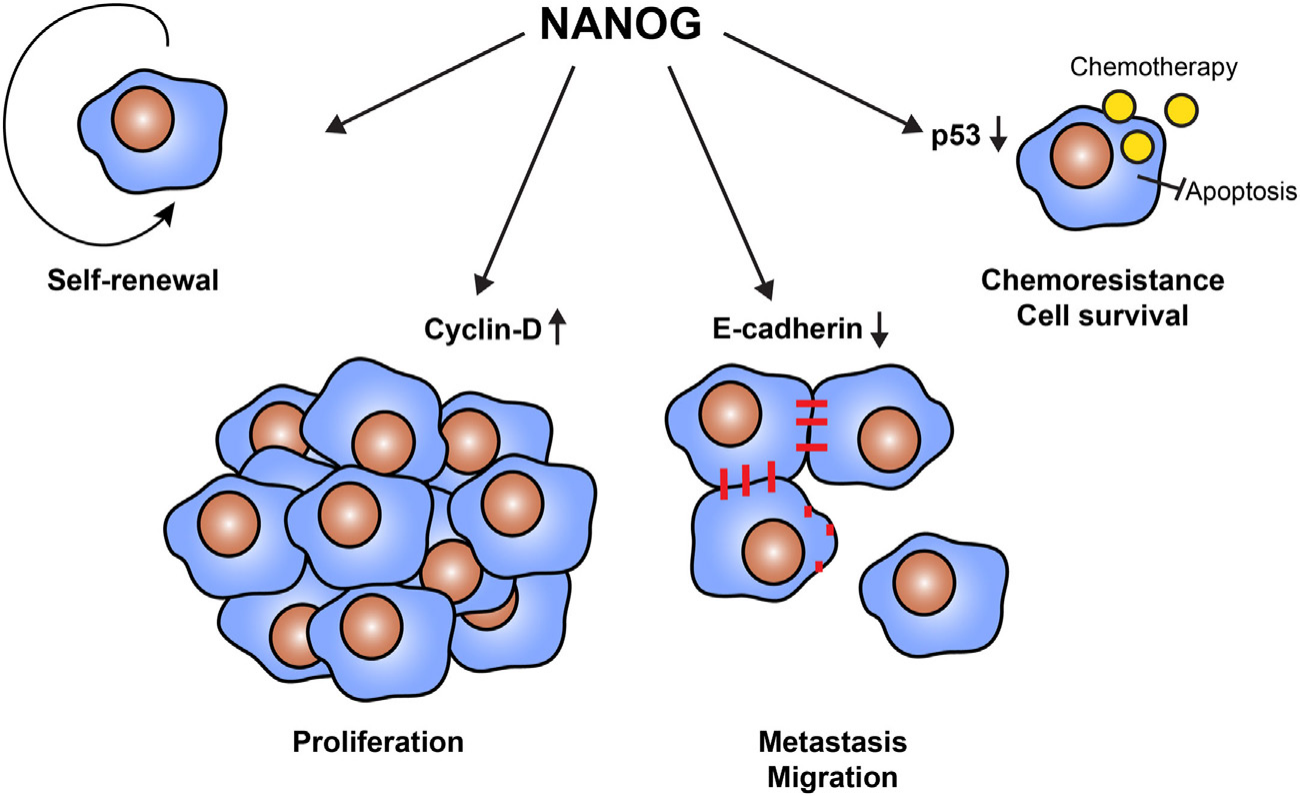
Cellular functions of NANOG in cancer stem cells (CSCs). The transcription factor NANOG is expressed by CSCs and has a variety of functions. NANOG is essential to maintain the self-renewal properties of CSCs. Furthermore, NANOG regulates cell proliferation *via* the interaction with cell cycle proteins, such as cyclin D. It also downregulates the expression of E-cadherin, enabling cells to detach and migrate to form distant metastases. Finally, NANOG promotes cell survival and resistance to therapy. NANOG interferes with the tumor suppressor p53, protecting cells from apoptosis.

The best way to kill CSCs is to target their unique proteins, not or low expressed by somatic cells (30). Candidates are the transcription factors OKT4a, SOX2, c-MYC, and KLF4, which also transform somatic cells into stem cells (iPS) (31). Most types of cancers express several of these transcription factors in a low percentage of cells (32–35), although some cancer types express only one or two of these transcription factors (36–38). Another candidate is the transcription factor NANOG, which regulates several cellular functions (Figure 1) (39). NANOG is required for maintaining stem cell properties and is re-expressed in a wide array of cancers (40–44). It furthermore promotes cell proliferation, migration, and metastasis, likely by downregulation of cell–cell interactions *via* E-cadherin (45) and control of cell cycle-related proteins (46). NANOG also renderers CSCs resistant to chemotherapy, for example, by inhibition of p53-mediated apoptosis (47). Expression of NANOG and its pseudo genes is low or absent in normal cells, making it an ideal therapeutic target (48–51).

### Expression of Stem Cell Factor NANOG in CSCs

Expression of NANOG in CSCs is detected in a variety of cancer types, including glioma (34), breast cancer (52), ovarian cancer (53), and lung cancer (54). The realization that the resistant cancer cells driving tumor recurrence after classical treatment express NANOG (34, 51, 55–60) have prompted the development of NANOG inhibitors. These inhibitors were remarkably successful in experimental models (42, 51, 55, 61–63). For example, activator-like effector nuclease (TALEN) was able to disrupt NANOG function in Hela cells *in vitro*, increasing chemosensitivity and reversing the epithelial-to-mesenchymal transition (64). In breast cancer cells, shRNA against NANOG reduced cell proliferation and migration (46). However, *in vivo* targeting of these compounds to CSCs will be a major challenge. Furthermore, wiping out NANOG by TALEN or shRNA (46, 64–66) will not suffice to eradicate cancer. Through a process known as plasticity, differentiated cancer cells become CSCs by re-expressing NANOG (67–71), which also occurs in non-pathogenic cells (72). NANOG inhibiting compounds like RNAi or small inhibitors, therefore, have to be given lifelong to keep the tumor encaged. Thus, although NANOG may be suitable to specifically target CSCs, there is a need for novel strategies that can also control new CSCs arising through plasticity.

## The Immune System as CSC Killer

Our body has developed a unique strategy to provide long-term protection from pathogens and cancerous cells: the immune system. Tumor infiltration by CD8 immune cells is associated with prolonged patient survival (73). However, these CD8^+^ T cells are often unable to eradicate the tumor because of inhibition by other, immunosuppressive cells in the tumor microenvironment, such as regulatory T cells (T_regs_). To reflect the strength of this immunosuppression, an “immunoscore” of a tumor can be computed as the ratio between immune stimulating and immune inhibiting cells (74). Immunotherapies aim to tip this balance toward immune activation. For example, immune checkpoint antibodies alleviate immunosuppression and increase patient survival, proving that the immune system can combat cancer.

However, can the immune system also prevent further cancer growth by attacking stem cell antigens? The first indication comes from a study where the immune system was activated by vaccinating with glioblastoma lysate of either normal cancer cells or CSCs (75). The quantity of stem cell antigens is much higher in the CSC lysate, which evokes a better immune response against the cancer. Other studies have shown that cancer patients as well as healthy subjects can have immunological memory against OCT4, a CSC transcription factor (76, 77). However, this memory response could not cure or prevent cancer. Full responses appear to require rejuvenation of the immune system by means of DCs loaded with tumor antigens (78). Nevertheless, the existence of immunological memory against CSC transcription factors is promising, as it suggests that it should be possible to generate long-lasting immune responses that control the CSCs driving tumor growth.

### The Tumor Uses Several Ways to Stop Immune-Mediated Killing

An important reason why existing anti-tumor immune cells fail to eradicate the tumor is active immunosuppression by the tumor microenvironment. Surviving tumors have escaped immunological clearance by paralyzing the immune system, exploiting several natural mechanisms by which the body can dampen immune reactions. These mechanisms include the attraction of immunosuppressive cells (T_regs_, myeloid-derived suppressor cells), induction of inhibitory cytokines (IL-10, TGF-β), reduction of MHC class I expression, expression of metabolic enzymes, such as indoleamine 2,3-dioxygenase and arginase (which breakdown either tryptophan or arginine, respectively) (79), and the PD-1–PD-L1 axis (80, 81). Mesenchymal stem cells (MSCs) that form part of the tumor microenvironment are able to inhibit the immune system. *In vitro*-cultured MSCs express NANOG and the expression of immune-modulatory role is dependent on NANOG. Destroying NANOG in activated MSCs in the tumor bed by the immune system will limit the immune inhibitors and is an added advantage (82). Yet another way to prevent immune attack is modification of the lipid pathways, resulting in a block of T-cell proliferation (83). Checkpoint antibodies relieve part of this inhibition and thereby prolong overall survival (84). However, as tumors use multiple immunosuppressive strategies, a single antagonist will be unable to fully abrogate immunosuppression and eradicate the tumor. It is, therefore, crucial to also look at other methods to tip the balance back in favor of anti-cancer immunity.

### Cytotoxic T Cells Need Target Molecules to Kill Tumor Cells

Another factor that may hamper T cell responses against CSCs is insufficient expression of HLA class I. Before cytotoxic T lymphocytes (CTLs) can kill a tumor cell, they must first recognize tumor antigens presented on HLA class I. Whereas, several studies reported reduced expression of HLA class I on CSCs, other studies nevertheless found a cytotoxic CD8 T cell response against CSCs [reviewed in Ref. (85)]. CSCs lacking HLA class I should be recognized by NK cells. In ovarian cancer, NK cells obtained from CD34 hematopoietic stem cells destroy cancer cells in a sphere assay, a classical way to culture CSCs (86).

**Figure 2:**
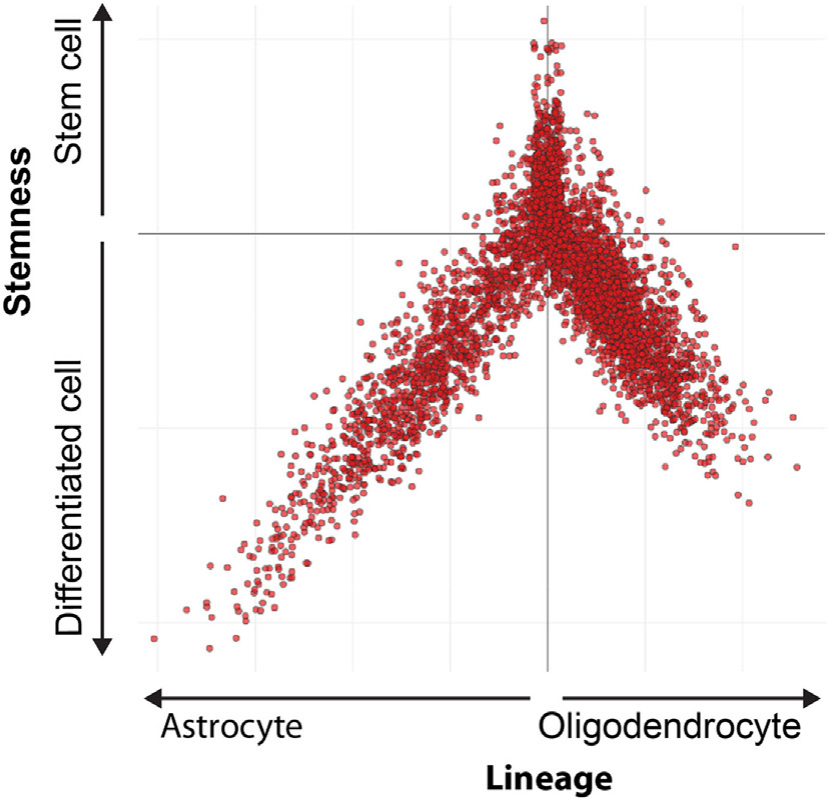
Differentiation hierarchy in oligodendrogliomas. Differentiation hierarchy based on differentiation scores (X) and stem cell scores (Y). Three distinct expression programs: oligodendrocyte (positive X, negative Y), astrocyte (negative X, negative Y), and stem cells (positive Y). Each dot represents a single cell. Reprinted by permission from Tirosh et al. (87).

**Figure 3:**
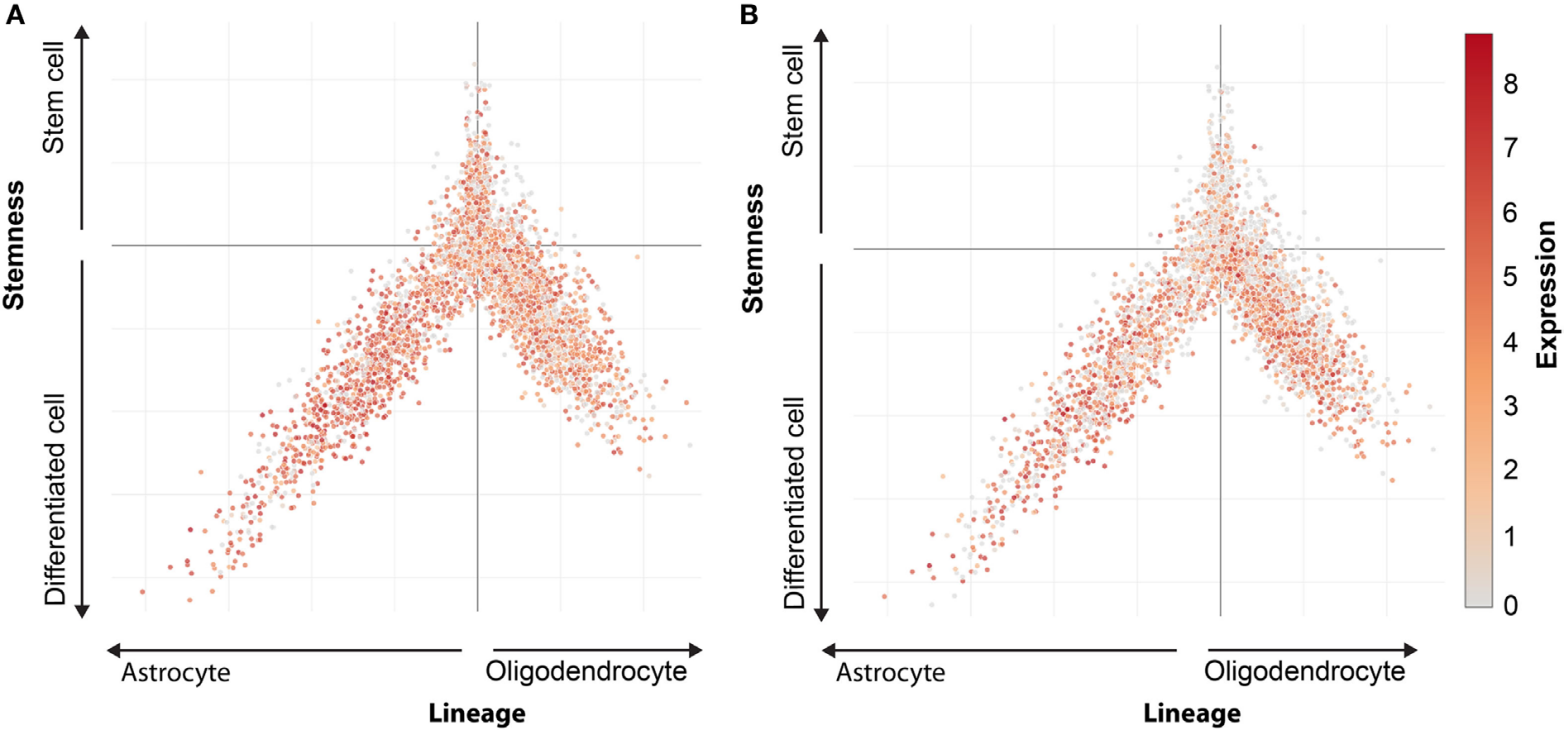
HLA expression in oligodendroglioma. Expression pattern of HLA-A **(A)** and HLA-B **(B)** in oligodendrocytes, astrocytes, and cancer stem cells. Every dot represents a single cells. Oligodendrocytes lineage (positive X, negative Y), astrocytes lineage (negative X, negative Y), and stem cells (positive Y). Expression level indicated by colors from gray (low expression) to red (high expression).

MHC class I expression in stem cell marker positive cells can be analyzed using single cell m-RNA sequencing. The Broad Institute performed single cell mRNA sequencing in oligodendrogliomas CSCs and their differentiated offspring (Figure 2) (87). A stem cell score and a lineage score was computed for each cell, enabling classification as stem cell, astrocyte, or oligodendrocyte. These data are available online (https://portals.broadinstitute.org/single_cell), and allowed us to investigate HLA class I expression on CSCs. Analysis showed that CSCs express HLA-A and B (Figure 3). Only a minority of CSCs and mature cancer cells did not express HLA class I.

Although these results suggest that it is feasible to evoke a CTL response against CSCs, HLA class I negative CSCs and mature cancer cells will escape elimination, forming a phenotype invincible by CD8^+^ T cells (88). These cells regain HLA class I during this dedifferentiation (89). This “soft” HLA class I loss can be resolved by administering IFN-γ or revering epigenetic modifications *via* HDAC inhibitors (90).

**Figure 4:**
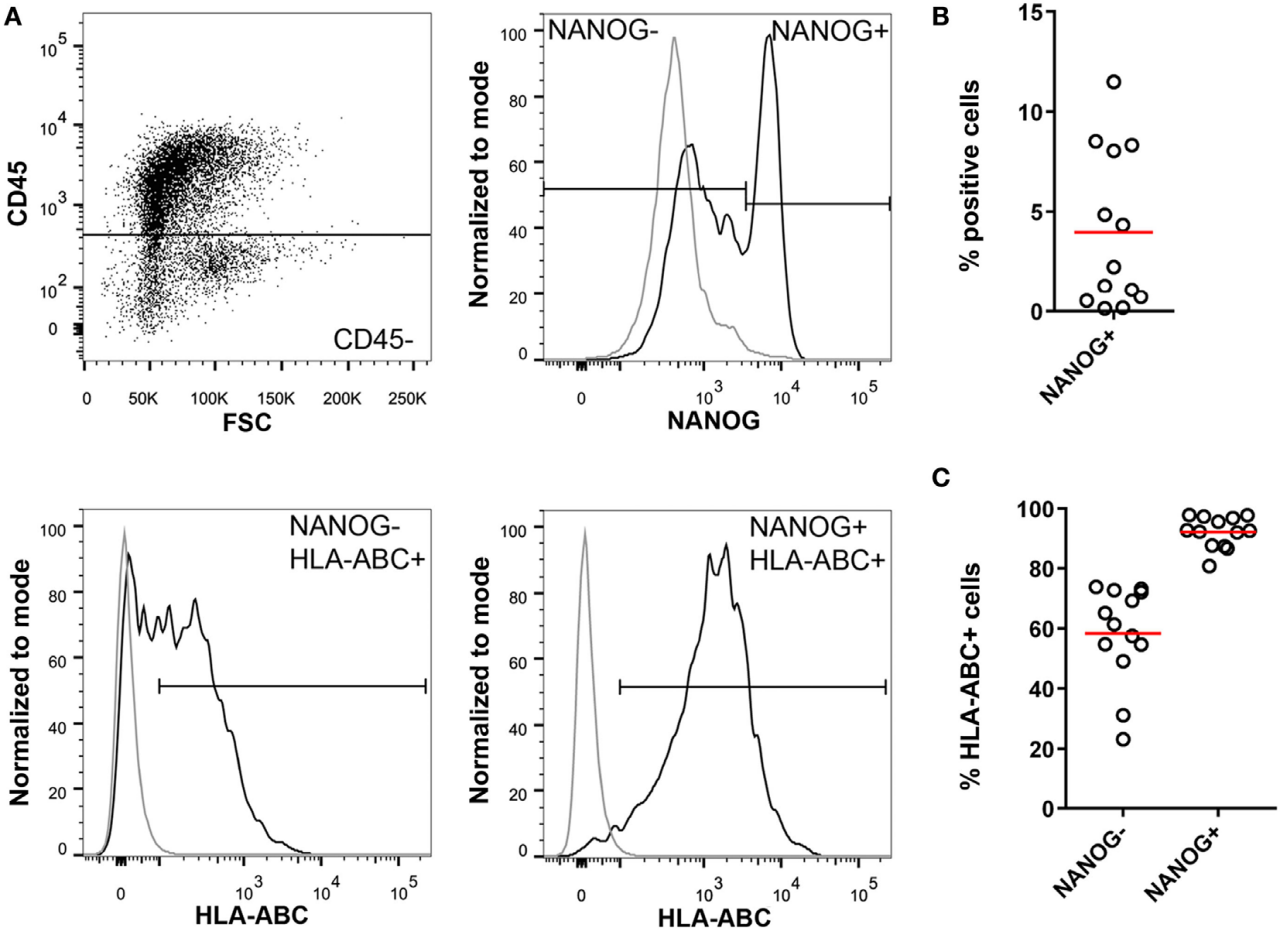
Cancer stem cells in high-grade serous ovarian cancer ascites. **(A)** Isolated mononuclear cells from ascites of ovarian cancer patients (*n* = 13) were stained for CD45, NANOG, and HLA-ABC. NANOG positivity was assessed in the CD45− population to exclude immune cells. NANOG− and NANOG+ cells were gated and HLA-ABC expression was analyzed in both populations. Gray line = isotype control; black line = sample. **(B)** Percentage of NANOG+ cells in ascites. **(C)** Percentage of HLA-ABC positive cells in the NANOG− and NANOG+ population. Red line indicates mean.

Importantly, we detected HLA class I on NANOG positive cells in ascites of ovarian cancer patients (Figure 4), whereas NANOG negative tumor cells were partially HLA class I negative. Apparently, the conversion of more mature cancer cells into CSCs (plasticity) requires epigenetic changes that allow re-expression of HLA class I. These findings suggest that CSCs expressing NANOG should be vulnerable for CD8 attack.

## DC Vaccination Against NANOG: CTL Memory as Guard Against CSCs

Dendritic cell vaccination could evoke cytotoxic T cells directed to CSCs and might induce immunological memory. Thus, this approach could not only eradicate existing CSCs but also provide the long-term ability to remove new CSCs arising through plasticity.

While over 100 trials have tried to boost the immune system by injecting autologous DCs loaded with aberrantly expressed or mutated proteins (neoantigens) (91, 92), only a few DC vaccination trials try to target CSCs. Several problems currently hamper the efficacy of these DC vaccination approaches against CSCs. First, they generally rely on isolation of autologous CSCs and loading of DCs with CSC lysates (75, 93, 94), a personal approach that is labor intensive and is hindered by the current lack of surface makers that can reliably isolate CSCs. Second, it remains unknown which neoantigens in the lysate elicit an immune response, and the number of these immunogenic neoantigens depends on the tumor. For example, colon cancer has five times more mutations than breast cancer (95). A high mutation rate increases the chance that neoantigens are presented to the immune system. But, neoantigens also need to bind with strong affinity to HLA class I molecules, as this determines the strength of the immune response (96). Current DC vaccination strategies against CSCs, therefore, have limited control over the initiated immune response and might not be suitable for every tumor type.

A more elegant way to eradicate CSCs is to load autologous DCs with specific peptides rather than tumor lysate, as this approach would provide better control over the immune response generated. Specifically, we propose loading DCs with NANOG peptides. Because cancer cells re-express NANOG when they regain stem cell properties, generation of immunological memory after DC vaccination against NANOG would help the immune system to cope with CSC plasticity.

We, therefore, used the NetMHCpan algorithm to determine which peptides from the NANOG protein can bind to HLA class I (97). Several HLA types are able to bind multiple NANOG peptides that could be presented to T cells (Table 1). In patients with HLA types that lack strong binders (e.g., HLA-A2), peptides from other CSC transcription factors, such as SOX2 or OKT4a, could be used (Table 2). As individuals generally have 4 to 6 different HLA class I molecules, cocktails of NANOG peptides that bind to the different HLA molecules should induce a potent immune response against CSCs. These results suggest that it is feasible to create DC vaccines tuned to the needs of individual patients.

**Table 1:** Predicted peptides from NANOG that bind to the most common HLA-A types.

Pos	HLA	Peptide	Score	% Rank	BindLevel
128	HLA-A*01:01	LSNILNLSY	0.925578	0.0772	≤SB
288	HLA-A*01:01	QTMDLFLNY	0.876012	0.122	≤SB
170	HLA-A*01:01	SAPTYPSLY	0.553264	0.4238	≤SB
275	HLA-A*01:01	NVIQQTTRY	0.402377	0.669	≤WB
233	HLA-A*01:01	NQAWNSPFY	0.263582	1.033	≤WB
285	HLA-A*01:01	STPQTMDLF	0.212155	1.2491	≤WB
27	HLA-A*01:01	VICGPEENY	0.125078	1.9183	≤WB
131	HLA-A*02:01	ILNLSYKQV	0.236804	1.29	≤WB
266	HLA-A*02:01	ALEAAGEGL	0.195211	1.5189	≤WB
124	HLA-A*02:01	QMQELSNIL	0.18792	1.5702	≤WB
297	HLA-A*02:01	SMNMQPEDV	0.150866	1.8894	≤WB
147	HLA-A*03:01	RMKSKRWQK	0.726852	0.1333	≤SB
288	HLA-A*03:01	QTMDLFLNY	0.521822	0.3469	≤SB
78	HLA-A*03:01	TSAEKSVAK	0.453863	0.4542	≤SB
160	HLA-A*03:01	KNSNGVTQK	0.229502	1.0894	≤WB
79	HLA-A*03:01	SAEKSVAKK	0.170143	1.3846	≤WB
143	HLA-A*03:01	FQNQRMKSK	0.150921	1.5158	≤WB
129	HLA-A*03:01	SNILNLSYK	0.103267	1.9548	≤WB
282	HLA-A*24:02	RYFSTPQTM	0.944242	0.0464	≤SB
135	HLA-A*24:02	SYKQVKTWF	0.808316	0.1963	≤SB
173	HLA-A*24:02	TYPSLYSSY	0.745947	0.2532	≤SB
205	HLA-A*24:02	TWSNQTQNI	0.563898	0.4686	≤SB
285	HLA-A*24:02	STPQTMDLF	0.452306	0.6464	≤WB
240	HLA-A*24:02	FYNCGEESL	0.356878	0.8489	≤WB
276	HLA-A*24:02	VIQQTTRYF	0.261095	1.1693	≤WB
292	HLA-A*24:02	LFLNYSMNM	0.247165	1.2256	≤WB
218	HLA-A*24:02	NHSWNTQTW	0.233086	1.2872	≤WB
213	HLA-A*24:02	IQSWSNHSW	0.194124	1.4867	≤WB
235	HLA-A*24:02	AWNSPFYNC	0.15336	1.7525	≤WB
157	HLA-A*24:02	NWPKNSNGV	0.129894	1.959	≤WB
275	HLA-A*26:01	NVIQQTTRY	0.945984	0.0055	≤SB
288	HLA-A*26:01	QTMDLFLNY	0.925988	0.0076	≤SB
50	HLA-A*26:01	TVSPLPSSM	0.436104	0.2259	≤SB
285	HLA-A*26:01	STPQTMDLF	0.420456	0.2396	≤SB
170	HLA-A*26:01	SAPTYPSLY	0.327707	0.3584	≤SB
233	HLA-A*26:01	NQAWNSPFY	0.135179	0.9734	≤WB
49	HLA-A*26:01	ETVSPLPSS	0.12096	1.0868	≤WB
268	HLA-A*26:01	EAAGEGLNV	0.114943	1.1405	≤WB
128	HLA-A*26:01	LSNILNLSY	0.100328	1.2837	≤WB
173	HLA-A*26:01	TYPSLYSSY	0.100053	1.2865	≤WB
246	HLA-A*26:01	ESLQSCMQF	0.094491	1.3466	≤WB
276	HLA-A*26:01	VIQQTTRYF	0.078035	1.5685	≤WB
166	HLA-A*26:01	TQKASAPTY	0.071117	1.6778	≤WB
169	HLA-A*26:01	ASAPTYPSL	0.059168	1.9468	≤WB

*Peptide length 9; rank threshold for strong binding peptide 0.500; rank threshold for weak binding peptide 2.000; SB, strong binding; WB, weak binding; predicted peptides were generated using NetMHCpan Version 4.0 (http://www.cbs.dtu.dk/services/NetMHCpan/)*.

**Table 2:** Predicted SOX2 and OCT4A peptides that bind to HLA-A.

Pos	HLA	Peptide	Score	% Rank	BindLevel
**SOX2**					
275	HLA-A*02:01	SMYLPGAEV	0.669833	0.2687	≤SB
131	HLA-A*02:01	LLAPGGNSM	0.42905	0.6673	≤WB
58	HLA-A*02:01	KMAQENPKM	0.375814	0.7882	≤WB
125	HLA-A*02:01	YTLPGGLLA	0.257884	1.1925	≤WB
236	HLA-A*02:01	ALGSMGSVV	0.203653	1.468	≤WB
216	HLA-A*02:01	YMNGSPTYS	0.146827	1.9276	≤WB

**OCT4A**					
268	HLA-A*02:01	GLEKDVVRV	0.719995	0.2203	≤SB
249	HLA-A*02:01	FLQCPKPTL	0.645456	0.2944	≤SB
72	HLA-A*02:01	GMAYCGPQV	0.492133	0.5388	≤WB
203	HLA-A*02:01	LLQKWVEEA	0.419854	0.6892	≤WB
163	HLA-A*02:01	TQADVGLTL	0.372758	0.795	≤WB
332	HLA-A*02:01	ALYSSVPFP	0.166148	1.7452	≤WB
93	HLA-A*02:01	SQPEGEAGV	0.164799	1.7569	≤WB

*Peptide length 9; rank threshold for strong binding peptide 0.500; rank threshold for weak binding peptide 2.000; SB, strong binding; WB, weak binding; predicted peptides were generated using NetMHCpan Version 4.0 (http://www.cbs.dtu.dk/services/NetMHCpan/)*.

## Why do we not have Memory T Cells?

Apparently, no immunity against NANOG exists in normal conditions or cancer would never develop. This suggests that while antigen-presenting cells may present NANOG, this presentation fails to trigger cytotoxic T cells. Several factors may account for this discrepancy. The strength of the immune response depends on the duration of antigen presentation, the affinity of peptides for HLA, and the abundance of peptides. The latter is evident from melanoma, where a normal protein (gp100) becomes immunogenic upon upregulation in malignant cells (98, 99). The peptides presented on the cell’s surface are continuously refreshed to reflect the internal intracellular pool of peptides. Peptides of less expressed proteins will typically be presented on HLA molecules in very low quantities that will be rapidly replaced for more abundant peptides. Moreover, both CSCs and DCs, the main presenters of antigenic peptides, are extremely rare. The chance that a DC encounters a CSC presenting NANOG peptides is, therefore, very low. Thus, immunity against NANOG requires a boost by either vaccination or by administration of DCs loaded with NANOG peptides. This approach would increase the number of DCs presenting CSC antigens, and also allows for the selection of suitable peptides based on the patient’s HLA type.

## Do *In Vitro* Raised CTL Kill CSCs?

To assess the potential of vaccination with NANOG-loaded DCs, it is crucial to investigate whether CTLs raised against NANOG *in vitro* are capable of killing CSCs. Current approaches come with limitations that make it difficult to assess the killing capacity of CTLs directed to CSCs. For example, recent data indicate that adherent cells only harbor a minute amount of CSCs, but it is hard to generate a cell line from tumor tissue.

To circumvent this problem, it is possible to grow spheroids from tumor tissue using low adhesive culture plates and special, serum-free media supplemented with the growth factors EGF and FGF (100–105). As these cultures go into apoptosis when the CSC is destroyed, as shown for the food supplements and NK cells (86), T cell killing of CSCs can be investigated by analyzing the shrinkage of spheroids co-cultured with *in vitro* raised T cells.

However, spheroids are rather simple structures that do not capture the complexity of an organ and are thus unsuitable to assess side effects. So far, we do not know if immunity to NANOG is detrimental for the patient. For melanoma patients, we sometimes observe vitiligo around a reactive mole, indicating that CTLs also attack normal melanocytes. Although *in vivo* experiments in xenotransplanted mice with siRNA against NANOG did not show adverse effects on normal mouse tissues. These results should be interpreted with caution as it is unknown whether siRNA also inhibits murine Nanog. Moreover, mice have a different MHC class I and thus present other peptides from murine Nanog.

### Organoids as a Method to Explore the Safety of Vaccination With NANOG-Loaded DC

Organoids from CSCs and normal stem cells would be a better approach to test both the efficacy and safety of CTLs raised against NANOG (106–109). Organoids are 3D structures grown from stem cells that are able to recapitulate organ structure and organ-specific cell types. They can be grown from embryonic or induced pluripotent stem cells, from organ-restricted adult stem cells, or from isolated CSCs. Organoid models currently exist for many different tissues, including brain, lung, small intestine, and kidney (110, 111). Living NANOG-expressing stem cells can be obtained by labeling cells with NANOG nanoflares and sorting the positive cells (112, 113). This enables to test DC vaccination against NANOG in a more natural 3D environment, solving the limitations of current *in vitro* and *in vivo* approaches.

## Conclusion

Reduction of the tumor mass, by either surgery or radiotherapy, is typically the first treatment for solid tumors. However, complete removal of the tumor mass is impossible in some cancer types, leading to disease recurrence when chemotherapy fails to eradicate remaining CSCs. DC vaccination against NANOG could solve this problem by specifically removing CSCs that fuel cancer growth. Importantly, these vaccines can be tuned to the patient’s HLA type to maximize response rates. If NANOG peptides cannot be presented by a patient’s HLA class I molecules, peptides against other stem cell transcription factor SOX2 or OKT4 may be used.

Despite the promise of this CSC targeting approach, durable immune responses will likely require a combination of DC vaccination with other therapies. Chemotherapy is not suitable in combination with DC vaccination, as it attacks hematopoietic cells in the bone marrow, making it difficult to recover enough autologous DCs for vaccination. To ensure an optimal immune response, inhibiting cells and soluble inhibitors should be removed as much as possible. Combining DC vaccination against NANOG with immune checkpoint inhibitors might help to overcome immunosuppressive mechanisms, and thereby potentiate the immune response directed against NANOG.

## Materials and Methods

### Patient Material

Ascites was obtained from stage III and IV high-grade serous ovarian cancer patients before start of the treatment. The study was carried out in accordance with the guidelines and regulation of the Radboudumc. The protocol was approved by the “Commissie Mensgebonden Onderzoek” (CMO Arnhem-Nijmegen). All subjects gave informed consent in accordance with the Declaration of Helsinki. Some samples were obtained before written consent was needed. Ascites was considered as waste material and only oral informed consent was necessary. All patients gave this oral informed consent to help future patients and were aware that it was not for their own benefit but for research purposes.

### Flow Cytometry Staining

Mononuclear cells from ascites were isolated using Ficoll-gradient centrifugation (Axis Shield) (114). Cells were washed with PBA (PBS/0.5% BSA/0.01% NaN_3_) and blocked for 10 min in PBA with 2% FcR blocking reagent (Miltenyi). Afterwards, cells were incubated for 30 min with a-CD45-V450 (1:50, BS Biosciences) and a-HLA-ABC-APC (1:4, Miltenyi). Subsequently, cells were fixed in 4% PFA and permeabilized with PBA containing 0.5% saponin (Sigma). Next, cells were incubated for 30 min with a-NANOG-PE (R&D Systems). Samples were measured on a BD FACS Verse and analyzed using FlowJo Version 10.

## Data Availability Statement

The datasets analyzed for this study can be found in the Broad Institute single cell portal (https://portals.broadinstitute.org/single_cell/study/oligodendroglioma-intra-tumor-heterogeneity#study-summary). All other relevant datasets for this study are included in the manuscript.

## Ethics Statement

Ascites was obtained from stage III and IV high-grade serous ovarian cancer patients before start of the treatment. The study was carried out in accordance with the guidelines and regulations of the Radboudumc. The protocol was approved by the “Commissie Mensgebonden Onderzoek” (CMO Arnhem-Nijmegen). All subjects gave informed consent in accordance with the Declaration of Helsinki. Some samples were obtained before written consent was needed. Ascites was considered as waste material and only oral informed consent was necessary. All patients gave this oral informed consent to help future patients and were aware that it was not for their own benefit but for research purposes.

## Author Contributions

CW and GS performed and analyzed immunological experiments. LM collected ascites and took care of informed consent. RT designed the study. RT and CW wrote the manuscript. JdV supervised the study. All authors reviewed the manuscript and approved the final version.

## Conflict of Interest Statement

The authors declare that the research was conducted in the absence of any commercial or financial relationships that could be construed as a potential conflict of interest.
